# Impact of radiotherapy to the primary tumor on the efficacy of pembrolizumab for patients with advanced urothelial cancer: A preliminary study

**DOI:** 10.1002/cam4.3445

**Published:** 2020-09-04

**Authors:** Hiroshi Fukushima, Toshiki Kijima, Shohei Fukuda, Shingo Moriyama, Sho Uehara, Yosuke Yasuda, Hajime Tanaka, Soichiro Yoshida, Minato Yokoyama, Yoh Matsuoka, Kazutaka Saito, Nobuaki Matsubara, Noboru Numao, Yasuyuki Sakai, Takeshi Yuasa, Hitoshi Masuda, Junji Yonese, Yukio Kageyama, Yasuhisa Fujii

**Affiliations:** ^1^ Department of Urology Tokyo Medical and Dental University Tokyo Japan; ^2^ Department of Breast and Medical Oncology National Cancer Center Hospital East Kashiwa Japan; ^3^ Department of Urology Cancer Institute Hospital Japanese Foundation for Cancer Research Tokyo Japan; ^4^ Department of Urology Saitama Cancer Center Saitama Japan; ^5^ Department of Urology National Cancer Center Hospital East Kashiwa Japan

**Keywords:** carcinoma, transitional cell, immunotherapy, pembrolizumab, radiotherapy, treatment outcome

## Abstract

Radiotherapy plus immune checkpoint inhibitors can potentially induce synergistic antitumor immune responses. However, little clinical evidence is established regarding their combination therapy. Here, we aimed to assess whether radiotherapy to the primary tumor impacts on the efficacy of pembrolizumab in advanced urothelial cancer. We retrospectively reviewed 98 advanced urothelial cancer patients receiving pembrolizumab in a second‐ or later‐line setting using our multicenter cohort. Patients were categorized according to a history of radiotherapy to the primary tumor: patients previously exposed to radiotherapy to the primary tumor (Radiotherapy group, 17 patients [17%]) and those not (Nonradiotherapy group, 81 patients [83%]). The associations of radiotherapy to the primary tumor with objective response and survival were evaluated. The Radiotherapy group showed a significantly higher objective response ratio than did the Non‐radiotherapy group (65% vs 19%; *P* < .001). The Radiotherapy group had a higher progression‐free survival rate compared with the Nonradiotherapy group (52% vs 28% at 12 months; *P* = .078), but statistical significance was not reached. The Radiotherapy group had a significantly higher overall survival rate compared with the Non‐radiotherapy group (77% vs 50% at 12 months; *P* = .025). From multivariate analysis, radiotherapy to the primary tumor was an independent predictor for longer overall survival (hazard ratio, 0.31; *P* = .032) along with Eastern Cooperative Oncology Group performance status ≤1 and the absence of visceral metastasis. Therefore, radiotherapy to the primary tumor may enhance the efficacy of pembrolizumab for patients with advanced urothelial cancer.

## Graphical abstract

Radiotherapy to the primary tumor was significantly associated with favourable therapeutic response and prognosis following pembrolizumab therapy in advanced urothelial cancer patients. Thus, radiotherapy to the primary tumor may enhance the efficacy of pembrolizumab, indicating the possibility that radiotherapy to the primary tumor in combination with pembrolizumab may be a promising therapeutic strategy for urothelial cancer.

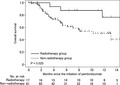

## 1 INTRODUCTION

Urothelial cancer patients who present with inoperable locally advanced and/or metastatic disease face poor prognosis. Their median duration of overall survival (OS) was reported to be 12‐15 months, despite favorable initial responses to induction systemic chemotherapy.^1^ The recent advent of immune checkpoint inhibitors (ICIs), which activate antitumor T cells by abrogating inhibitory signaling, has changed a paradigm in therapeutic strategies for advanced urothelial cancer.^2^ According to the KEYNOTE‐045 trial, pembrolizumab, an antiprogrammed cell death 1 (PD‐1) agent, yielded more favorable outcomes compared with chemotherapy in a second‐ or later‐line setting.^3^ However, only approximately 20% of patients achieved objective response and more than 40% showed progressive disease (PD) after pembrolizumab therapy,^3^ reflecting the unmet need to identify therapeutic strategies to convert nonresponders to responders.

Accumulating data showed that radiotherapy stimulates systemic antitumor immune activity.^4^, ^5^ Radiotherapy promotes T‐cell receptor repertoire diversification through increased release of tumor‐associated antigens.^6^ Ionizing radiation‐damaged DNA activates the cyclic GMP‐AMP synthase (cGAS) stimulator of interferon genes (STING) pathway, leading to increased secretion of type I interferon.^7^ Damage‐associated molecular patterns, which characterize immunogenic cell death, trigger strong antitumor immunity through secreting T‐cell‐attracting chemokines.^4^ In preclinical studies, radiotherapy showed a synergistic antitumor immune activity in combination with ICIs through these responses, resulting in enhanced abscopal effects, where nonirradiated lesions regress following radiation.^5^, ^8^, ^9^, ^10^ Therefore, given that the primary tumor generally has a large tumor burden, we hypothesized that advanced urothelial cancer patients who previously received radiotherapy to the primary tumor would have an enhanced antitumor immune activity with pembrolizumab. To verify this hypothesis, we investigated the effect of radiotherapy to the primary tumor on the efficacy of pembrolizumab for patients with advanced urothelial cancer.

## 2 MATERIALS AND METHODS

### 2.1 Patients

This retrospective study included 98 advanced urothelial cancer patients (unresectable cT4 and/or lymph node/visceral metastasis) treated with pembrolizumab as a second‐ or later‐line therapy between January 2018 and August 2019 using a multicenter cohort composed of four tertiary hospitals. A fixed dose of pembrolizumab (200 mg/body) was intravenously infused every 3 weeks. All patients were pathologically diagnosed with urothelial carcinoma. The study protocol was approved by the ethical committee of each institution. Because of the retrospective design of the study, written informed consent was waived. The following variables were assessed in the present study: age, sex, Eastern Cooperative Oncology Group performance status (ECOG PS), primary site, presence of lymph node/visceral metastasis, visceral metastatic sites, prior definitive surgery, first‐line chemotherapy regimen, line of pembrolizumab, a duration of pembrolizumab therapy, time since most recent chemotherapy, immune‐related adverse events (irAEs), a history of radiotherapy to the primary tumor, chemotherapy concurrent with radiotherapy to the primary tumor, hemoglobin, and serum C‐reactive protein (CRP). The cutoffs of hemoglobin and serum CRP were set to the values used in previous studies.^3^, ^11^ irAE grade was determined according to the Common Terminology Criteria for Adverse Events, version 5.0.^12^

### 2.2 Endpoints

The endpoints were objective response rate (ORR), progression‐free survival (PFS), and OS. Radiologists at each institution evaluated response to pembrolizumab based on the Response Evaluation Criteria in Solid Tumors (RECIST), version 1.1.^13^ Either complete response (CR) or partial response (PR), which was determined by best overall response, was regarded as objective response. PFS was calculated as the interval from the initiation of pembrolizumab therapy to disease progression, death, or censored at the last follow‐up. OS was calculated as the interval from the initiation of pembrolizumab therapy to death or censored at the last follow‐up.

### 2.3 Statistical analysis

Distributions of categorical or continuous variables were compared among groups using the chi‐square test or Mann‐Whitney U test, respectively. Variables associated with objective response were evaluated by the logistic regression analyses. PFS and OS curves were depicted using the Kaplan‐Meier methods followed by comparisons using the log‐rank test. Associations between variables OS were assessed by the Cox proportional hazards analyses. To generate a reduced multivariate model, backward elimination of variables was iteratively conducted in multivariate analyses comprised of variables with *P* < .10 on univariate assessment. Statistical studies were done using JMP 9.0 (SAS Institute Inc, Cary, NC, USA). Two‐tailed *P* < .05 was regarded as statistical significance.

## 3 RESULTS

### 3.1 Patient characteristics

Clinical parameters of the total patients are shown in Table 1. The median (interquartile range [IQR]) age was 70 (65‐75) years. Seventy‐six (78%) and 54 (55%) patients had lymph node and visceral metastases, respectively. Lung, bone, and liver metastases were present in 38 (39%), 20 (20%), and 17 (17%) patients, respectively. The primary site was the bladder in 57 (58%) patients. Before the diagnosis of advanced urothelial cancer, 52 (53%) patients had a history of curative surgery (radical nephroureterectomy, n = 29; radical cystectomy, n = 22; and partial cystectomy, n = 1). Seventy‐one (72%) patients were previously treated with cisplatin‐based chemotherapy.

**TABLE 1:** Patient characteristics

Variables		Total n (%) or median (IQR)	Radiotherapy to the primary tumor		*P* value
			Yes, n (%) or median (IQR)	No, n (%) or median (IQR)	
No. of patients		98 (100)	17 (17)	81 (83)	
Age (yr)		70 (65‐75)	71 (63‐79)	70 (66‐75)	.48
Sex	Male	76 (78)	16 (94)	60 (74)	.072
	Female	22 (22)	1 (6)	21 (26)	
ECOG PS	0‐1	87 (89)	16 (94)	71 (88)	.44
	≥ 2	11 (11)	1 (6)	10 (12)	
Primary site	Bladder	57 (58)	16 (94)	41 (51)	<.001
	UUT	41 (42)	1 (6)	40 (49)	
Lymph node metastasis	No	22 (22)	2 (12)	20 (25)	.25
	Yes	76 (78)	15 (88)	61 (75)	
Visceral metastasis	No	44 (45)	9 (47)	35 (43)	.46
	Yes	54 (55)	8 (53)	46 (57)	
Visceral metastatic sites	Lung	38 (39)	6 (35)	32 (40)	.75
	Bone	20 (20)	4 (24)	16 (20)	.73
	Liver	17 (17)	2 (12)	15 (19)	.50
Prior definitive surgery	No	46 (47)	11 (66)	35 (43)	.11
	Yes	52 (53)	6 (35)	46 (57)	
Line of pembrolizumab	Second	70 (71)	12 (71)	58 (72)	.29
	Third	20 (20)	5 (29)	15 (19)	
	Fourth	8 (8)	0 (0)	8 (10)	
Duration of pembrolizumab therapy (months)		3 (1‐7)	6 (2‐11)	3 (1‐6)	.028
Time since most recent chemotherapy	<3 months	47 (48)	8 (47)	39 (48)	.93
	≥3 months	51 (52)	9 (53)	42 (52)	
First‐line chemotherapy regimen	Cisplatin based	71 (72)	13 (76)	58 (72)	.58
	Carboplatin based	22 (22)	4 (24)	18 (22)	
	Others	5 (5)	0 (0)	5 (6)	
Hemoglobin	<10.0 g/dL	36 (37)	7 (41)	29 (36)	.68
	≥10.0 g/dL	62 (63)	10 (59)	52 (64)	
Serum CRP	<5.0 mg/L	40 (41)	7 (41)	33 (41)	.97
	≥5.0 mg/L	58 (59)	10 (59)	48 (59)	
Any grade irAE	No	67 (68)	9 (53)	58 (72)	.13
	Yes	31 (32)	8 (47)	23 (28)	
Grade 3‐4 irAE	No	90 (92)	15 (88)	75 (93)	.55
	Yes	8 (8)	2 (12)	6 (7)	

Note

Abbreviations: CRP, C‐reactive protein; CRT, chemoradiotherapy; ECOG PS, Eastern Cooperative Oncology Group performance status; IQR, interquartile range; irAE, immune‐related adverse events; UUT, upper urinary tract.

### 3.2 Radiotherapy to the primary tumor

Radiotherapy to the primary tumor was delivered in 17 (17%) patients for a median (IQR) of 14 (5‐22) months prior to initiating pembrolizumab therapy (details in Table S1). Patients were categorized into two groups: patients previously exposed to radiotherapy to the primary tumor (Radiotherapy group, 17 [17%]) and those not (Non‐radiotherapy group, 81 [83%]). In the Radiotherapy group, 13 (76%) patients received concurrent chemotherapy (cisplatin, n = 11; gemcitabine, n = 1; and tegafur‐gimeracil‐oteracil potassium capsules [S‐1], n = 1; Table S1). Radiotherapy to the primary tumor was delivered to nine (53%) patients with definitive intent and eight (47%) with palliative intent. Of the nine patients treated with definitive intent, five received radiotherapy to the primary tumor as a neoadjuvant therapy prior to definitive surgery^14^, ^15^, ^16^ and four received radiotherapy to the primary tumor definitively.

### 3.3 Clinical outcomes of pembrolizumab

Pembrolizumab was administered in 70 (71%), 20 (20%), and 8 (8%) patients in second, third‐, and fourth‐line settings, respectively. The best overall response to pembrolizumab was as follows: CR, 2 (2%) patients; PR, 24 (24%); stable disease (SD), 24 (24%); and PD, 48 (49%). The ORR of the total cohort was 27%. Any grade and grade 3‐4 irAEs were observed in 31 (32%) and 8 (8%) patients, respectively. Pembrolizumab therapy was terminated due to disease progression or irAEs in 38 (39%) patients. The median (IQR) duration of pembrolizumab therapy was 3 (1‐7) months. Patients with objective response (median [IQR], 8 [4‐13]) had a significantly longer duration of pembrolizumab therapy compared with those with SD and PD (median [IQR], 2 [1‐4]; *P* < .001). At a median (IQR) follow‐up duration of 5 (2‐11) months, 48 (49%) and 66 (67%) patients were free from progression and alive, respectively. The 12‐month PFS and OS rates were estimated at 34% and 56%, respectively.

### 3.4 Relationship between radiotherapy to the primary tumor and other clinical parameters

The associations of radiotherapy to the primary tumor with other clinical parameters are shown in Table 1. The primary site was in the bladder in a significantly higher proportion of the Radiotherapy group compared with the Non‐radiotherapy group (94% vs 51%; *P* < .001), reflecting the fact that radiotherapy‐based treatment to the primary tumor is established in muscle‐invasive bladder cancer including bladder preservation therapy.^14^, ^15^, ^16^, ^17^ The Radiotherapy group had a significantly longer duration of pembrolizumab therapy (*P* = .028). Statistical difference was not present in age, sex, ECOG PS, presence of lymph node metastasis, presence of visceral metastasis, visceral metastatic sites, prior definitive surgery, line of pembrolizumab, time since most recent chemotherapy, first‐line chemotherapy regimen, hemoglobin, serum CRP, and rates of any grade and grade 3‐4 irAE of pembrolizumab between the Radiotherapy and Non‐radiotherapy groups.

### 3.5 Impact of radiotherapy to the primary tumor on therapeutic response to pembrolizumab

In each group, the best overall response to pembrolizumab was as follows: CR, 1 (6%) patient; PR, 10 (59%); SD, 1 (6%); and PD, 5 (29%) in the Radiotherapy group and CR, 1 (1%); PR, 14 (17%); SD, 23 (28%); and PD, 43 (53%) in the Non‐radiotherapy group. The Radiotherapy group had a significantly higher ORR than did the Non‐radiotherapy group (65% vs 19%; *P* < .001). From multivariate logistic regression analysis, radiotherapy to the primary tumor (odds ratio, 8.34; 95% confidence interval, 2.67‐28.75; *P* < .001) was independently associated with objective response (Table 2).

**TABLE 2:** Logistic regression analysis for objective response

Variables		Univariate	Multivariate		
		*P* value	OR	95% CI	*P* value
Age	< 65 yr	.058	0.36	0.12‐1.08	.066
	≥ 65 yr				
Sex	Females	.13			
	Males				
ECOG PS	0‐1	.51			
	≥2				
Primary site	Bladder	.077			
	UUT				
Lymph node metastasis	No	.32			
	Yes				
Visceral metastasis	No	.54			
	Yes				
Prior definitive surgery	No	.72			
	Yes				
Line of pembrolizumab	Second	.82			
	Third/Fourth				
Time since most recent chemotherapy	<3 months	.83			
	≥3 months				
First‐line chemotherapy regimen	Carboplatin based/Others	.55			
	Cisplatin based				
Radiotherapy to the primary tumor	No	<.001	ref.		<.001
	Yes		8.34	2.67‐28.75	
Definitive‐intent radiotherapy to the primary tumor	No	.21			
	Yes				
Hemoglobin	≥10.0 g/dL	.46			
	<10.0 g/dL				
Serum CRP	<5.0 mg/L	.27			
	≥5.0 mg/L				

Note

Abbreviations: CI, confidence interval; CRP, C‐reactive protein; ECOG PS, Eastern Cooperative Oncology Group performance status; OR, odds ratio; UUT, upper urinary tract.

### 3.6 Prognostic value of radiotherapy to the primary tumor

**FIGURE 1:**
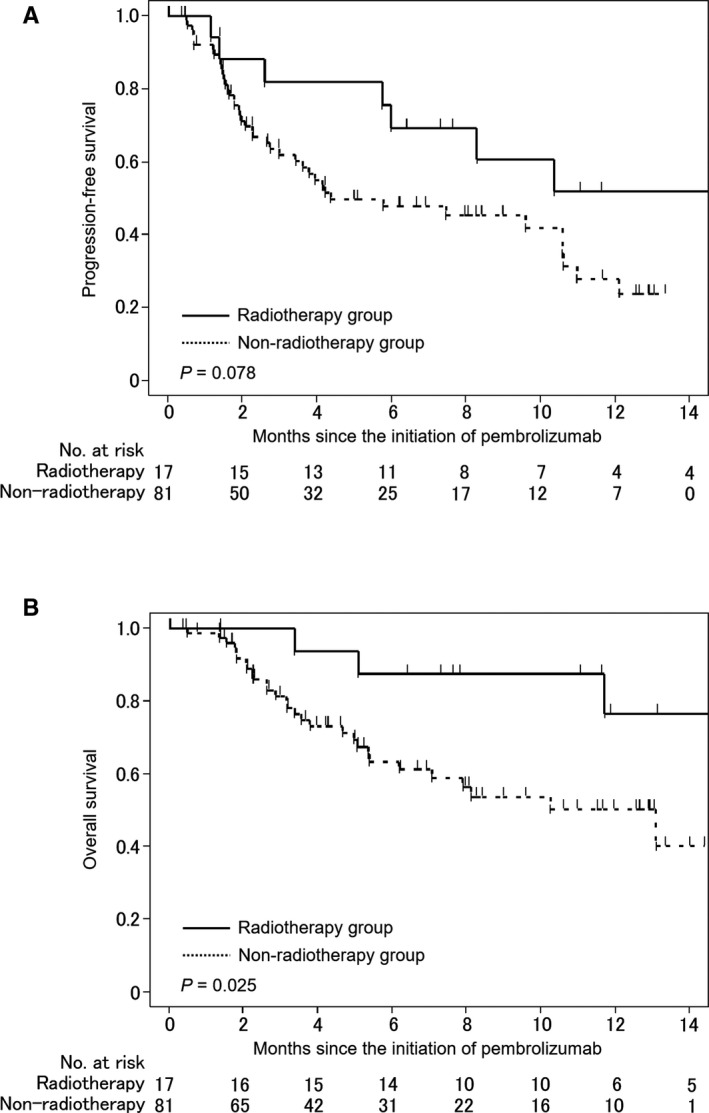
Progression‐free (A) and overall (B) survival curves of patients based on their history of radiotherapy to the primary tumor

The Radiotherapy group had a higher PFS rate than did the Non‐radiotherapy group (52% vs 28% at 12 months; *P* = .078; Figure 1A), but the statistical significance was not reached. The Radiotherapy group showed a significantly higher OS rate compared with the Non‐radiotherapy group (77% vs 50% at 12 months; *P* = .025; Figure 1B). From univariate analysis, ECOG PS, primary site, presence of visceral metastasis, radiotherapy to the primary tumor, and serum CRP were significantly related to OS (Table 3). From multivariate analysis, radiotherapy to the primary tumor was an independent predictor for longer OS (hazard ratio, 0.31; 95% confidence interval, 0.07‐0.91; *P* = .032), along with ECOG PS ≤ 1 (*P* < .001) and the absence of visceral metastasis (*P* = .017; Table 3).

**TABLE 3:** Cox proportional hazards analysis for overall survival

Variables		Univariate	Multivariate		
		*P* value	HR	95%CI	*P* value
Age	<65 yr	.65			
	≥65 yr				
Sex	Females	.13			
	Males				
ECOG PS	0‐1	<.001	ref.		<.001
	≥2		5.01	2.05‐11.15	
Primary site	Bladder	.014			
	UUT				
Lymph node metastasis	No	.14			
	Yes				
Visceral metastasis	No	.009	ref.		.017
	Yes		2.52	1.17‐6.02	
Prior definitive surgery	No	.75			
	Yes				
Line of pembrolizumab	Second	.87			
	Third/Fourth				
Time since most recent chemotherapy	<3 months	.71			
	≥3 months				
First‐line chemotherapy regimen	Carboplatin based/Others	.48			
	Cisplatin based				
Radiotherapy to the primary tumor	No	.014	ref.		.032
	Yes		0.31	0.07‐0.91	
Definitive‐intent radiotherapy to the primary tumor	No	.42			
	Yes				
Hemoglobin	≥10.0 g/dL	.074			
	<10.0 g/dL				
Serum CRP	<5.0 mg/L	.019			
	≥5.0 mg/L				

Note

Abbreviations: CI, confidence interval; CRP, C‐reactive protein; ECOG PS, Eastern Cooperative Oncology Group performance status; HR, hazard ratio; UUT, upper urinary tract.

## 4 DISCUSSION

We revealed a significant association of radiotherapy to the primary tumor with a higher ORR in the present study. Surprisingly, the ORR of patients previously treated with radiotherapy to the primary tumor was 65%, which is approximately three times higher than that of the pembrolizumab group in the KEYNOTE‐045 trial.^3^ Moreover, radiotherapy to the primary tumor was an independent predictor for longer OS. To our knowledge, this study is the first to reveal that radiotherapy to the primary tumor was significantly associated with favorable therapeutic efficacy and prognosis following pembrolizumab therapy in advanced urothelial cancer patients. Our results indicate that radiotherapy to the primary tumor plus pembrolizumab may be a promising therapeutic strategy for urothelial cancer. Our preliminary findings should be confirmed by future and ongoing prospective studies evaluating outcomes of radiotherapy in combination with ICIs in urothelial cancer.^18^

Many researchers explore possible ways to improve therapeutic responses to ICIs. Radiotherapy has been focused upon due to its synergistic effect with ICIs.^4^, ^5^ In a previous preclinical study, radiotherapy up‐regulated the cell surface expression of programmed cell death ligand 1 (PD‐L1) in bladder cancer cells, in whose immune microenvironment PD‐L1 blockade may be more effective.^19^ Moreover, radiotherapy can facilitate tumor‐associated antigen release,^6^ activation of the cGAS‐STING pathway,^7^ and immunogenic cell death.^4^ Preclinical studies showed that these responses provoked by radiotherapy could enhance antitumor immunity in combination with ICIs.^5^, ^8^, ^9^, ^10^ Our results can be explained by this mechanism. In the KEYNOTE‐001 trial, a history of thoracic and/or extracranial radiotherapy was a significant predictor for longer PFS and OS after pembrolizumab therapy for patients with nonsmall‐cell lung cancer,^20^ which corresponded with our results. Because the primary tumor generally has a large tumor burden, our results suggest that antitumor immunity might be activated effectively by radiotherapy to the primary tumor in combination with pembrolizumab.

A previous preclinical study indicated that immune responses stimulated by radiotherapy were nondurable. Dovedi et al reported that PD‐L1 blockade with concomitant radiotherapy showed a better survival compared with PD‐L1 blockade 7 days after radiotherapy using colon cancer mouse models.^8^ They also showed that PD‐1 up‐regulation in CD4+/CD8+T cells was observed 1 day after radiotherapy but not 7 days after radiotherapy, suggesting that nondurable PD‐1 expression induced by radiotherapy can limit the efficacy of PD‐L1 blockade.^8^ In our cohort, radiotherapy to the primary tumor was performed for a median of 14 months prior to initiating pembrolizumab therapy. In the KEYNOTE‐001 trial, radiotherapy was conducted for a median of 9.5 months prior to initiating pembrolizumab therapy.^20^ Thus, the time gap from radiotherapy to pembrolizumab therapy suggests that durable immune responses may be induced as a consequence of immune priming by radiotherapy, resulting in improved efficacy of pembrolizumab. Further preclinical examinations are necessary to clarify whether immunological memory can be generated through immune priming by radiotherapy.

In the present study, chemotherapeutic agents were concurrently used with radiotherapy to the primary tumor in 76% of the Radiotherapy group. Chemotherapeutic agents including cisplatin have been reported to be a potential inducer of immunogenic cell death.^21^, ^22^ Moreover, recently preclinical studies have shown that cisplatin enhanced the abscopal effects of PD‐1 blockade‐based radioimmunotherapy.^9^, ^23^ Because cisplatin was mostly used as a chemotherapeutic agent concurrent with radiotherapy to the primary tumor in our cohort, immune‐modulatory potentials of cisplatin might have facilitated an antitumor immune activity provoked by radiotherapy.

There are several limitations to the present study. First, due to its retrospective nature and small sample size, our findings may be affected by selection bias. The number of patients in the Radiotherapy group was only 17, indicating that our preliminary results need confirmation by larger studies. Because radiotherapy‐based therapeutic strategies are established in bladder cancer as bladder preservation therapy, radiotherapy to the primary tumor was mostly conducted in patients with bladder cancer.^14^, ^15^, ^16^, ^17^ Nevertheless, clinical parameters other than primary site were statistically similar between the Radiotherapy and Non‐radiotherapy groups. In addition, the ORR (19%) and PFS and OS rates (28% and 50% at 12 months, respectively) of the Non‐radiotherapy group were comparable to those after pembrolizumab therapy in the KEYNOTE‐045 trial (ORR, 21.1%; PFS and OS rates at 12 months, 16.8% and 43.9%, respectively), providing reassurance that our results were not being driven by selection bias. Second, the duration of response was not evaluated in the present study because it was not available in our database. Third, this study did not use iRECIST,^24^ which was recently proposed to assess therapeutic responses to ICIs. It is not yet prevalent in clinical practice possibly due to its complicated evaluation method, and thus RECIST version 1.1 was used in the present study.^13^ However, assessment of PD by RECIST version 1.1 can be more premature compared with iRECIST, which may result in underestimation of PFS especially in the Radiotherapy group. A nonsignificant trend of improved PFS for the Radiotherapy group may be explained by this. Finally, the safety of radiotherapy to the primary tumor in combination with pembrolizumab was not fully evaluated. In the present study, radiotherapy to the primary tumor was not associated with higher rates of any grade and grade 3‐4 irAE. However, the follow‐up period of the present study was relatively short, and thus its safety should be confirmed in longer follow‐up studies.

In conclusion, radiotherapy to the primary tumor was significantly associated with favorable therapeutic response and prognosis following pembrolizumab therapy in advanced urothelial cancer patients. Thus, radiotherapy to the primary tumor may enhance the efficacy of pembrolizumab. Although our findings are preliminary, they raise the possibility that a combination of radiotherapy to the primary tumor and pembrolizumab may be a promising treatment against urothelial cancer.

## CONFLICTS OF INTEREST

YF received honoraria for lectures from MSD KK, a subsidiary of Merck & Co., Inc, Kenilworth, NJ, USA; All other authors have no conflict of interest; All authors had full access to all of the data in the study and had final responsibility for the decision to submit for publication.

## AUTHOR CONTRIBUTION

Hiroshi Fukushima, Toshiki Kijima, Soichiro Yoshida, and Yasuhisa Fujii contributed to conception and design of study. Hiroshi Fukushima, Toshiki Kijima, Nobuaki Matsubara, Noboru Numao, and Yasuyuki Sakai contributed to acquisition of data. Hiroshi Fukushima, Toshiki Kijima, Hajime Tanaka, Soichiro Yoshida, and Yasuhisa Fujii contributed to analysis and/or interpretation of data. Hiroshi Fukushima and Yasuhisa Fujii contributed to drafting the manuscript. Toshiki Kijima, Shohei Fukuda, Shingo Moriyama, Sho Uehara, Yosuke Yasuda, Hajime Tanaka, Soichiro Yoshida, Minato Yokoyama, Yoh Matsuoka, Kazutaka Saito, Nobuaki Matsubara, Takeshi Yuasa, Hitoshi Masuda, Junji Yonese, and Yukio Kageyama revising the manuscript critically for important intellectual content. All authors have read and approved the submitted version.

## ETHICAL APPROVAL

M2019‐059 (Tokyo Medical and Dental University), 2019‐1143 (Cancer Institute Hospital), 997 (Saitama Cancer Center), and 2019‐205 (National Cancer Center Hospital East).

## Supporting information

Table S1

## Data Availability

Research data are not shared.
