# Supplementary material for: Impact of radiotherapy to the primary tumor on the efficacy of pembrolizumab for patients with advanced urothelial cancer: A preliminary study
Source: Cancer Med. 2020 Sep 4;9(22):8355–63. doi: 10.1002/cam4.3445 (PMC7666746; doi:10.1002/cam4.3445)
Supplement: Supplementary file 1 — Table S1 [file CAM4-9-8355-s001.docx]

**Table S1 Regimens of radiotherapy to the primary tumor**

| Regimen | No. of patients (%) |
| --- | --- |
| 40 Gy with cisplatin | 11 (65) |
| 50 Gy with gemcitabine | 1 (6) |
| 60 Gy with tegafur-gimeracil-oteracil potassium capsules (S-1) | 1 (6) |
| 30 Gy | 1 (6) |
| 39 Gy | 1 (6) |
| 60 Gy | 1 (6) |
| 66 Gy | 1 (6) |
